# Discovery of a new subfamily expands the catalytic versatility of vanillyl alcohol oxidases

**DOI:** 10.3389/fmicb.2026.1769237

**Published:** 2026-02-03

**Authors:** Nils Weindorf, Tobias Rapsch, Willem J. H. van Berkel, Dirk Tischler

**Affiliations:** 1 Department of Microbial Biotechnology, Faculty for Biology and Biotechnology, Ruhr University Bochum, Bochum, Germany; 2 Laboratory of Food Chemistry, Wageningen University & Research, Wageningen, Netherlands

**Keywords:** enzyme discovery, over-oxidation, oxidative deamination, phylogeny, vanillyl alcohol oxidase

## Abstract

Flavoenzymes of the 4-phenol oxidoreductase family are versatile biocatalysts that catalyze the oxidation of a wide variety of phenol derivatives to alcohols, aldehydes, ketones or alkenes. The promiscuous FAD-dependent vanillyl alcohol oxidases from *Penicillium simplicissimum* (*Ps*VAO) and *Diplodia corticola* (*Dc*VAO) have been described to catalyze the oxidative deamination of *p*-hydroxybenzylamines, giving rise to valuable flavor compounds, but starting from *p*-alkyl substituted phenols, the ketones are usually not accessible as these oxidases preferably stop at chiral benzylic alcohols. Here we took a closer look into the fungal VAO family with the aim to identify new members that can perform this deamination reaction and also the overoxidation of benzylic alcohols to ketones at a sufficient rate for application. Phylogenetic and amino acid cluster analysis revealed one clade that differed significantly in the constitution of the active site, while maintaining residues essential for catalysis. From this clade, five candidates were chosen for investigation, which revealed that VAO from *Paecilomyces variotii* (*Pv*VAO) showed promising activities with vanillylamine and 4-(1-amino)ethylphenol, especially above pH 9.0, while also offering the ability to perform the overoxidation of *p*-alkyl substituted phenols toward ketones. Hence, the identified *Pv*VAO offers two reaction routes toward benzylic ketones.

## 1 Introduction

Vanillyl alcohol oxidases (EC: 1.1.3.38) are fungal oxidases that are proposed to be involved in the natural degradation of lignin. As covalently flavinylated oxidoreductases, 8α-N3-histidyl-FAD in this case, VAOs belong to the VAO/PCMH flavoprotein superfamily, which is characterized by a conserved FAD binding domain, while the substrate binding domain is less conserved, and more precisely VAOs belong to the subfamily of 4-phenol oxidizing enzymes of this superfamily (Ewing et al., 2017a). To date, a small number of VAOs has been described, with the first being isolated and characterized from the *ascomycetous* fungus *Penicillium simplicissimum* (*Ps*VAO), and is also the most characterized with regards to structure and mechanism (Benen et al., 1998; De Jong et al., 1992; Fraaije and van Berkel, 1997; Fraaije et al., 1998; Mattevi et al., 1997). *Ps*VAO was shown to form tetramers of catalytically active dimers, which is conferred by a loop that is exclusive to the fungal VAOs as it is not found for the bacterial counterparts, the eugenol oxidases, which only form dimers (Ewing et al., 2016). Later, a putative VAO from *Byssochlamys fulva* V107 had been reported however, no sequence and structure were determined (Furukawa et al., 1999). The VAO of *Diplodia corticola* was described to differ from the earlier described *Ps*VAO by introducing two active site mutations that allowed the enzyme to efficiently convert di-*o*-substituted phenolic substrates, which can also occur in the degradation of hardwood lignin, that are not converted by *Ps*VAO (Eggerichs et al., 2023a). Four additional VAOs that feature similar active site and high sequence similarity with *Ps*VAO were also described recently in the context of engineering a VAO for the chemoenzymatic synthesis of 2-aryl thiazolines from 4-hydroxy benzaldehydes (Zhang et al., 2024). However, to our knowledge, these VAOs were not further characterized.

Foundational studies of *Ps*VAO revealed that this enzyme has a broad pH optimum with an optimum around pH 10 (De Jong et al., 1992). Stopped-flow kinetics with several substrates then established that at pH 7.5, the first step of the *Ps*VAO reaction, i.e., the reduction of the flavin cofactor by substrate, is rate-limiting in catalysis (Fraaije and van Berkel, 1997). Subsequently, it was established that the strictly conserved Y108, Y503, and R504 residues of the P-cluster form a phenolate binding pocket and thus are responsible for substrate activation (Ewing et al., 2017b; Mattevi et al., 1997). With certain substrates a more peculiar pH dependence of *Ps*VAO was observed. In the reaction with *p*-cresol and *p*-creosol, a pH optimum around pH 7.5 was found (Fraaije et al., 1998; van den Heuvel et al., 2000b). With these short chain alkylphenols, formation of a covalent adduct between substrate and flavin N5 was stimulated at high pH, resulting in suicide inhibition (Fraaije et al., 1998; Mattevi et al., 1997; van den Heuvel et al., 2001). Quantum chemical modeling suggested that the short side chain of *p*-creosol enabled D170, which is crucial for the redox properties of *Ps*VAO (van den Heuvel et al., 1998; van den Heuvel et al., 2000b), to initiate adduct formation, and that this abortive reaction is for steric reasons not possible with longer 4-alkylphenols (Gygli, PhD thesis Wageningen University 2018). Another interesting pH dependence was observed in the reaction of *Ps*VAO with vanillylamine (van den Heuvel et al., 2001). With this substrate, the enzyme appeared to be hardly active with vanillylamine at pH 7.5, but the activity increased dramatically above pH 9, reaching an apparent turnover rate of 1.0 s^−1^ at pH 10.5. Spectral analysis clearly established that the reaction with vanillylamine proceeded through the initial formation of vanillylimine and resulted in nearly 100% yield of the final product vanillin. The unusual pH dependence observed with vanillylamine was proposed to be related to the binding properties and/or activation of the amine substrate, but no attempts were made to address this issue in further detail.

Overall, VAOs were shown to be quite versatile biocatalysts however, unlike as it has been described for their bacterial counterparts (Eggerichs et al., 2024), the diversity of the active sites of the described VAOs and the thereby expected differences in the substrate scopes of VAOs has not been matched yet. Therefore, we were intrigued to take another look at the family of fungal VAOs to identify VAOs with differing active sites and possibly different substrate scopes, to achieve more insight in their applicability toward the production of valuable aromatic aldehydes and ketones. We produced five new VAOs from a new subfamily of the fungal VAOs, of which four could be produced as active proteins with distinct substrate scopes that differ from the ones reported for *Ps*VAO and *Dc*VAO.

## 2 Methods

### 2.1 Phylogenetic comparison

The sequences of known VAOs were used as a template for a Blastp search^1^ (Altschul et al., 1990). Retrieved results were combined and duplicates removed. Sequences of known bacterial eugenol oxidases, the bacterial counterparts of fungal VAOs, were added to the sequences as an outgroup. A multiple sequence alignment (MSA) in MEGA 11 was performed using the ClustalW algorithm (Tamura et al., 2021; Thompson et al., 1994). The MSA was then used to construct a phylogenetic tree using the maximum likelihood method. Based on known residues that form the active site of VAOs, A^2^CA (Eggerichs et al., 2024) was then used to compare the constitution of the active site throughout the protein family. Interesting candidates were chosen accordingly.

### 2.2 Cloning of VAO genes

Genes encoding for the selected VAOs were obtained as *Escherichia coli* codon-optimized synthetic gene fragments from Twist Biosciences (South San Francisco, CA, USA). Cloning into the pET28a expression vector for *E. coli* was done using Gibson method (Gibson et al., 2009). Primers for the pET28a backbone and the respective gene fragments with compatible overhangs were designed accordingly for insertion between the NdeI and NotI restriction sites (see Supplementary material, section 2) and used for amplification by PCR. The PCR was performed using the PrimeSTAR Max 2X Master Mix by Takara Bio (Saint-Germain-en-Laye, France). Initial denaturation of templates was done at 98 °C for 2 min, followed by 30 cycles of 20 s denaturation at 98 °C, annealing for 15 s at 60 °C, elongation for 10 s per 1 kb at 72 °C, and a final elongation for 5 min at 72 °C. Successful amplification of the fragments was confirmed *via* agarose gel electrophoresis and afterwards, a DpnI digestion was performed. The obtained fragments were then used for Gibson assemblies, using the HiFi-Assembly Mix and protocol from NEB (Frankfurt am Main, Germany), followed by heat shock transformation into *E. coli* DH5α and selection on LB-agar plates containing 35 mg/L kanamycin. Colony-PCR and Sanger-sequencing using T7 and T7term primers were used to identify successful colonies. Plasmids of positive hits were isolated and transformed into *E. coli* NiCo21(DE3) for heterologous protein production.

### 2.3 Protein production

Production of *Dc*VAO was done as described previously (Eggerichs et al., 2023a). The other VAOs were produced by heterologous protein production in *E. coli* NiCo21(DE3). For this, precultures were grown overnight at 37 °C. Precultures were then used to inoculate 1 L cultures in TB medium containing 35 mg/L kanamycin. The cells were grown at 37 °C, 120 rpm in a New Brunswick Innova 42R shaker (Hamburg, Germany) until an OD_600_ of 1.0. Then, the temperature was reduced to 20 °C and IPTG was added to a final concentration of 250 μM. Incubation was continued overnight. Cells were then harvested *via* centrifugation at 4,000×*g* using a Beckman Coulter Avanti JXN-26 (Brea, United Kingdom) using a JLA-8.1000 rotor. The sedimented cells were then washed with 30 mL 50 mM potassium phosphate pH 7.5 and centrifuged again. The washed cells were stored at −20 °C for further use.

### 2.4 Protein purification

Approximately 4.5 g wet cells were mixed with 30 mL of 50 mM potassium phosphate pH 7.5, 500 mM NaCl (Buffer A) and then placed on ice until thawed. The cells were then lysed on ice using a Bandolin SonoPlus (Berlin, Germany) sonificator equipped with a MS 72 probe. The total cell lysis procedure involved 30 cycles of 10 s sonication and 10 s of resting, at an amplitude of 30%. The obtained lysates were centrifuged for 45 min at 50,000×*g* using a Beckman Coulter Anvanti JXN-26 (Brea, United Kingdom) and a JA-25.50 rotor. The obtained supernatant was filtered using a 0.2 μm filter for protein purification using Ni-NTA affinity chromatography using a Cytiva ÄKTA start FPLC (Marlborough, MA, USA) and a 1 mL His-Trap HP column. After washing, the lysate was loaded at a flow rate of 2 mL/min. The column was then washed with 10 column volumes of Buffer A, followed by washing with 10% Buffer B (50 mM potassium phosphate pH 7.5, 500 mM NaCl, 500 mM imidazole), followed by elution of the target protein with 100% Buffer B. The obtained proteins were subjected to buffer exchange using a Cytiva PD-10 column to 50 mM potassium phosphate pH 7.5. UV/Vis spectra were recorded using an Agilent Cary 60 spectrophotometer, and proteins were diluted with glycerol to a final concentration of 50% glycerol for storage at −20 °C. Determination of the protein concentration was done based on the covalently bound cofactor as described (Jin et al., 2007), yielding the amount of FAD-loaded protein.

### 2.5 Substrate screening using a xylenol orange-based assay

Screening of the substrate scopes of the new VAOs was done using a xylenol orange-based assay, utilizing the stochiometric production of hydrogen peroxide, allowing for substrate-independent quantification, as it was described earlier for *Ps*VAO, EUGO and variants thereof (Ewing et al., 2018). Reactions contained 2 mM of the tested substrates in 50 mM potassium phosphate pH 7.5 and 50 nM of the respective enzyme, with a total volume of 100 μL. End-point measurements in a 96-well format were performed at 3, 6, and 9 min by mixing 20 μL of the reaction mixtures with 180 μL of xylenol orange solution (625 mM H_2_SO_4_, 69.8 mg/L FeSO_4_, 100 μM xylenol orange) and quantified by recording the absorbance at 560 nm using a Tecan Infinite Pro plate reader (Männedorf, Switzerland). Enzyme activities were calculated according to the corresponding slopes, hydrogen peroxide standards and the amount of used VAO. For determination of the pH-dependence of *Pv*VAO in deamination reactions, the XO-assay was adjusted accordingly.

### 2.6 Product identification using GC–MS

For the identification of the products formed by the VAOs, reactions were performed in 400 μL 50 mM potassium phosphate pH 7.5 with 2 mM of the respective substrates and 1 μM of the respective VAOs. Reactions in 1.5 mL tubes were incubated overnight at 25 °C while shaking at 750 rpm on an LLG Thermix Pro 2 (Meckenheim, Germany). Reactions were stopped by the addition of 400 μL of ethyl acetate, followed by rapid mixing for 60 s. After phase separation, the organic phase was transferred into a new 1.5 mL tube and dried by the addition of MgSO_4_. Afterwards, the samples were analyzed via GC–MS using a Shimadzu QP2020 NX (Kyoto, Japan) and a CS-Chromatographie FS Supreme 5 ms 60 m column. The method consisted of a split temperature of 300 °C, a temperature gradient from 50 to 250 °C at 10 °C/min, column flow of 1.3 mL/min and EI of 1.25 keV. Identification of substrates and products was done *via* comparison of mass spectra to the NIST-2017 library.

## 3 Results

### 3.1 Phylogenetic comparison of VAOs and selection of candidates

**Figure 1:**
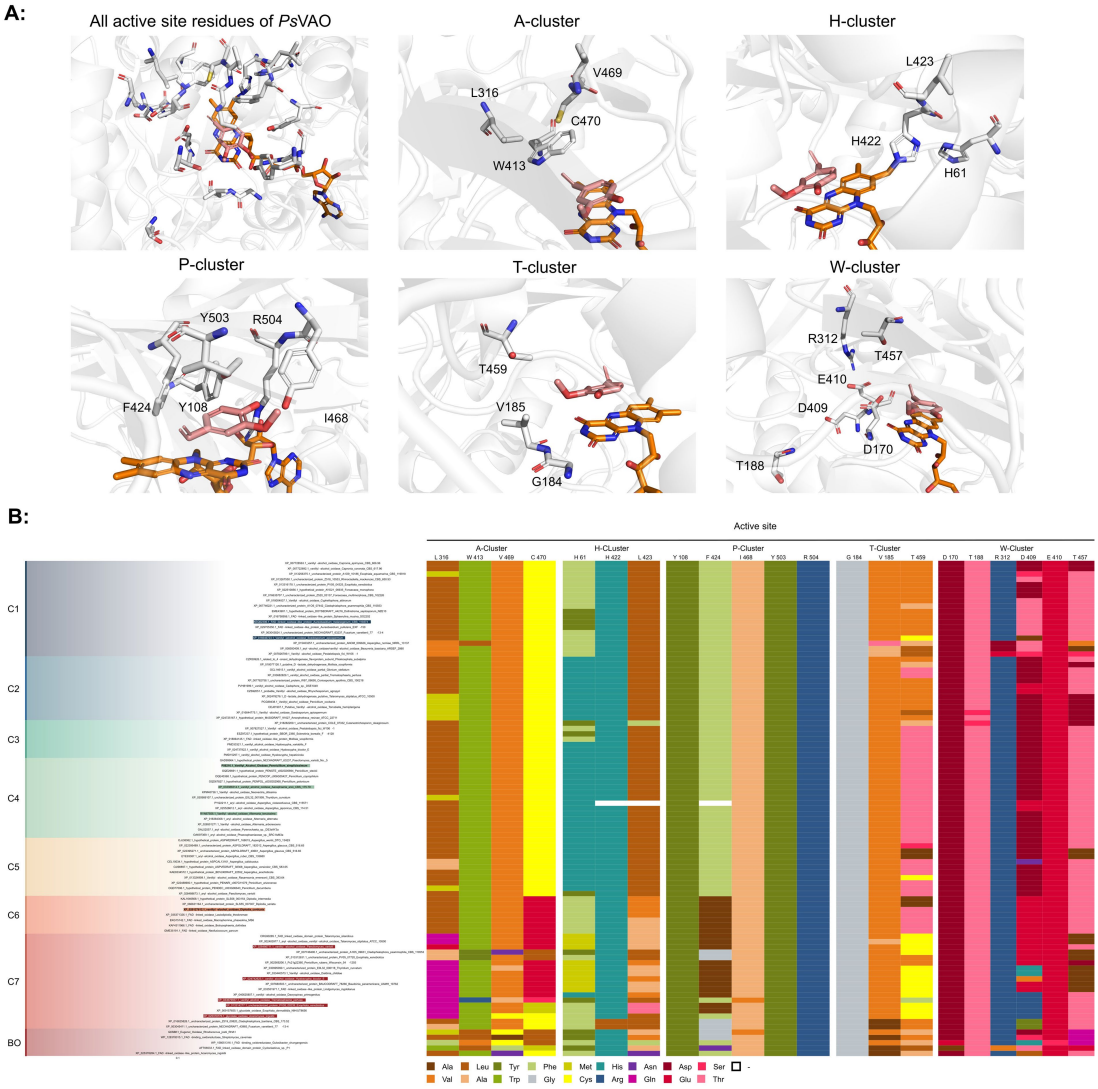
**(A)** Representation of the active site of *Ps*VAO (PDB: 2VAO). All first shell residues as well as the residues of each cluster are shown. **(B)** Output of A^2^CA (modified). The generated MSA and phylogenetic tree were used to highlight the diversity of fungal VAOs in their active sites, based on the five proposed clusters, the A-cluster, the H-cluster, the P-cluster, the T-cluster, and the W-cluster. *Ps*VAO (NCBI: P56216.1) was used as a reference and the respective positions within the sequence are indicated. The found residues in other VAOs are indicated by color (see legend). A bacterial outgroup (BO) of known eugenol oxidases was also included in the comparison.

For the generation of the phylogenetic tree, sequences of already known VAOs were used to perform a BLASTp search for fungal homologs (Benen et al., 1998; Eggerichs et al., 2023a; Zhang et al., 2024). In addition, putative VAOs that could not be produced as active proteins in *Komagataella phaffii* were also included (Gygli and van Berkel, 2017). The results were combined, duplicates removed, and three eugenol oxidases were included as an outgroup (Alvigini et al., 2021; Eggerichs et al., 2025; Jin et al., 2007; Weindorf et al., 2025). After construction of a phylogenetic tree and performing a multiple sequence alignment, and based on knowledge of the constitution of the active site, A^2^CA was then used to gain a deeper look into the diversity of the active site (Altschul et al., 1990; Eggerichs et al., 2024; Tamura et al., 2021; Thompson et al., 1994). As described earlier, the active site of VAOs and EUGOs can be divided into five clusters, based on their proposed interactions with either the substrate or the covalently bound FAD cofactor (Eggerichs et al., 2024). As a reference, the sequence of *Ps*VAO was used (NCBI: P56216.1). For *Ps*VAO, the A-cluster consists of four residues (L316, W413, V469, and C470) for coordination of the *p*-substituent of phenolic substrates, the H-cluster consisting of three residues (H61, H422, and L423) for covalent binding of the FAD cofactor, the P-cluster consisting of five residues (Y108, F424, I468, Y503, and R504) for coordination of the phenolic moiety of the substrates, the T-cluster consisting of three residues (G184, V185, and T459) forming the gate between the substrate tunnel and the active site, and the W-cluster consisting of six residues (D170, T188, R312, D409, E410, and T457) involved directly in catalysis as well as in binding and activating water molecules in hydroxylation reactions. For further investigation of the VAOs, focus was put on the diversity of their respective active sites as it was assumed that these would be a promising starting point to identify candidates with differences in the active site and possible changes in their substrate scopes. Figure 1A shows the active site of *Ps*VAO in its entirety and highlights the residues of each cluster with regards to their positioning toward the substrate and FAD cofactor. Figure 1B shows the output of A^2^CA, linking the sequence motif of the active site for each member of the VAOs to its position in the phylogenetic tree, emphasizing inter-clade conservation of certain residues and conservation across the family, which is not directly obtained through a phylogenetic tree and a MSA alone, thus adding a layer of information on top of these.

Based on the phylogenetic tree alone, the outgroup of the bacterial enzymes can clearly be differentiated from the VAOs. The VAOs can be divided into two subfamilies, the first one spanning over six clades (C1–C6) and the second one containing only clade C7. The division into two fungal subfamilies was reasoned by C1–C6 featuring the characteristic octamerization loop, which has been shown to be responsible for the formation of tetramers of dimers for *Ps*VAO (Ewing et al., 2016), while C7 lacks this loop, which is also the case for the bacterial counterparts (see Supplementary Figures S1, S2). Here, it is also worth noting that the constitution of this loop is also rather conserved within the respective clades.

The first subfamily of the VAOs contains the already described *Ps*VAO (C4), *Dc*VAO (C6), *Aa*VAO (C4), *At*VAO (C4), *Sa*VAO (C2) and *Am*VAO (C1). For C3 and C5 and for the second subfamily (C7), no enzymes have been characterized yet. Focusing a bit more onto the constitution of the active sites, key features for the clades can be observed. Clade C1 differs from the other VAOs in the H-, T-, and W-cluster, where in the H-cluster H61 (*Ps*VAO-numbering), proposed to be involved in autocatalytic covalent flavinylation (Fraaije et al., 2003; Fraaije et al., 2000), is replaced by a phenylalanine or tryptophan. In the T-cluster of this clade, T459 is mostly replaced by a valine or other aliphatic residue, like it is seen for some of the bacterial counterparts, while in the W-cluster, D408 is mostly replaced by a threonine. Clade C2 is closer to the other VAOs, with the change in the T-cluster of T459 to a valine or other aliphatic residue as mentioned for clade C1 is the only major difference. Concerning the amino acid residues that restrict the *o*-substituents of the phenolic substrates of VAOs (P- and T-cluster), clades C1–C5 are more restricted than clade C6 due to the presence of F424 (*Ps*VAO-numbering). Except for having F424 in the P cluster replaced by a less sterically demanding alanine, the constitution of clade C6 also differs from the other clades by having in the A cluster C470 exchanged by a glutamate, introducing an acid function in the active site, by having H61 in the H cluster replaced by a phenylalanine and L468 exchanged by a sterically less demanding isoleucine or valine, and by having D408 in the W cluster replaced by the larger glutamate.

The second subfamily (clade C7) clearly differs from the rest of the VAOs and is most close to clade C6 from the first subfamily (Figure 1). In the A-cluster of C7, the glutamate found in C6 is mostly retained, while also harboring a tyrosine and the commonly found cysteine among the other VAOs. Further, L316 is mostly replaced by a glutamine, introducing a more hydrophilic group into the A-cluster. The H-cluster is also closest to clade C6 however, H61 can be replaced by a methionine. The P-cluster of C7 is also similar to clade C6 however, F424 can be replaced by a glycine instead of an alanine, which is also seen for the bacterial counterparts. The T-cluster of C7 differs by having T459 mostly exchanged by a cysteine. The W-cluster of C7 also differs by either having D408 replaced by a glutamate, as found in clade C6, or by an arginine, histidine or tyrosine. Overall, the second subfamily shows a higher diversity than the first subfamily. Intrigued by this diversity, five VAOs, *Ai*VAO (NCBI: XP_025378284.1), *Ex*VAO (NCBI: XP_013314277.1), *Hi*VAO (NCBI: XP_024742623.1), *Pv*VAO (NCBI: XP_028486711.1), and *Tp*VAO (NCBI: XP_033679937.1) from the second subfamily were selected for further investigation (see Supplementary material, sequences in section 2, active sites Supplementary Figure S3).

### 3.2 Enzyme production and initial characterization

Of the five selected VAOs from the second subfamily, four could be produced as soluble and FAD-loaded proteins in *E. coli* NiCo21(DE3) using TB medium, while *Tp*VAO was produced as a cofactor-less protein where constitution with FAD was not possible. The others could be produced as FAD-loaded proteins in sufficient yields for investigation, without further optimization (see Supplementary material). It is worth noting that *Pv*VAO presented itself as an orange protein at the beginning of the protein purification and underwent a color change from orange to yellow during the first washing step to remove unbound proteins from the used column.

**Figure 2:**
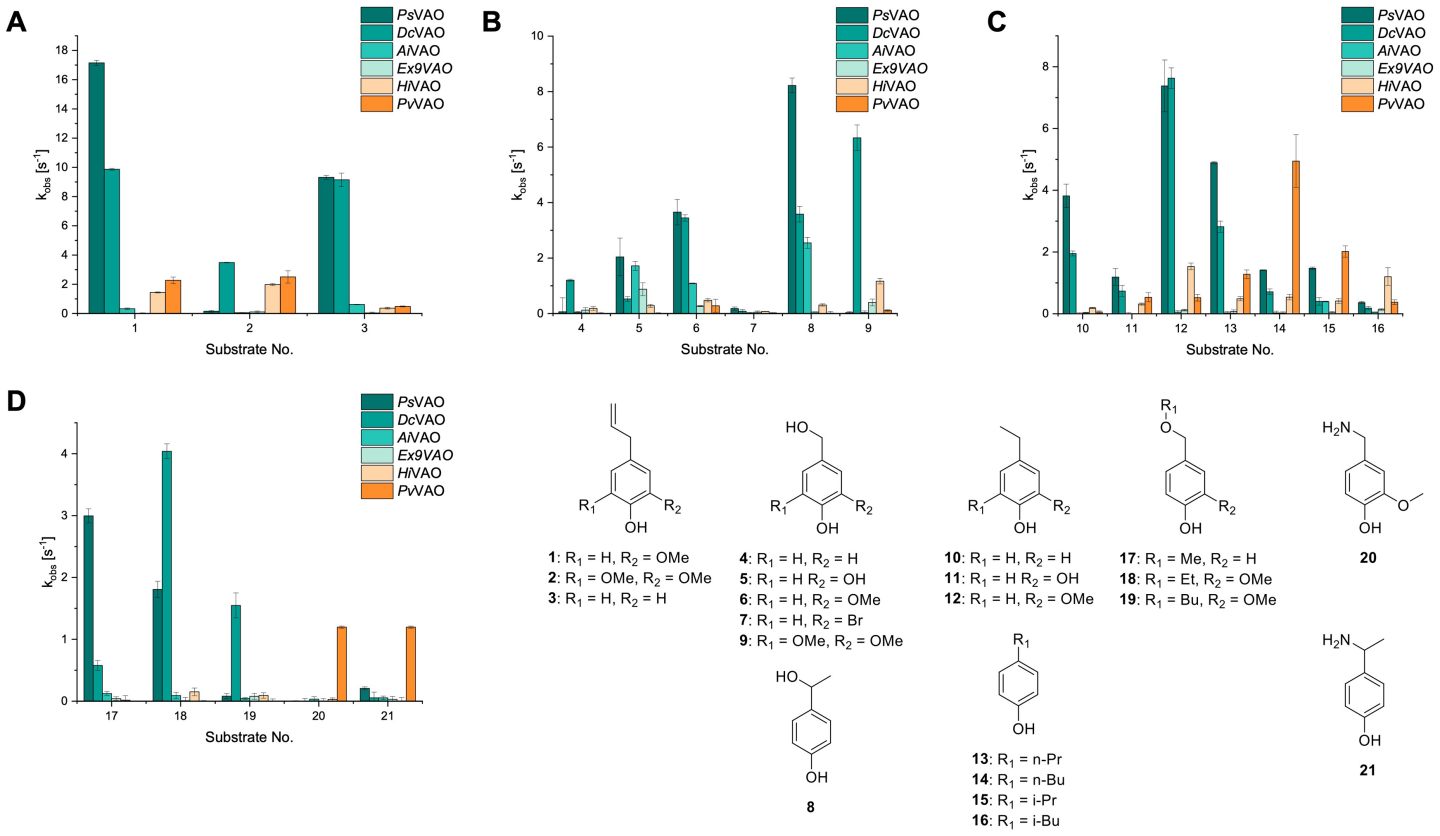
Activity data for *Ps*VAO, *Dc*VAO, *Ai*VAO, *Ex*VAO, *Hi*VAO, and *Pv*VAO. **(A)** *k*_obs_ (s^−1^) of the respective VAOs for substrates **1–3** for γ-hydroxylation reactions, **(B)**: *k*_obs_ (s^−1^) of the respective VAOs for substrates **4–9** for oxidation reactions of benzylic alcohols, **(C)**: *k*_obs_ (s^−1^) of the respective VAOs for substrates **10–16** for α-hydroxylation and/or dehydrogenation reactions of 4-alkylphenols, and **(D)** *k*_obs_ (s^−1^) of the respective VAOs for substrates **17–21** for the oxidative dealkylation of phenolic ethers and oxidative deamination of phenolic amines. The activity data was obtained using a xylenol orange-based assay as described in 2.5.

The xylenol orange-based screening assay (Eggerichs et al., 2024; Ewing et al., 2018) was performed to assess the activity of the four newly produced VAOs with a set of substrates that cover the reactions that can be catalyzed by VAOs (Figure 2). Overall, the new VAOs were active on most of the tested substrates but showed lower specific activities (*k*_obs_ values) than the already described *Ps*VAO and *Dc*VAO.

A typical reaction of VAOs is the γ-hydroxylation of 4-allylphenols (see Figure 2A). *Ps*VAO efficiently converted eugenol (**1**) (17.2 s^−1^) and chavicol (**3**) (9.3 s^−1^), while *Dc*VAO converted next to **1** (9.9 s^−1^) and **3** (9.2 s^−1^) also 4-allyl-2,6-dimethoxyphenol (**2**) (3.5 s^−1^). *Ex*VAO did not show any activity toward these substrates. *Ai*VAO, *Hi*VAO and *Pv*VAO performed these γ-hydroxylation reactions, but the *k*_obs_ values for **1** (0.3 s^−1^, 1.4 s^−1^, and 2.3 s^−1^), **2** (0 s^−1^, 2.0 s^−1^, and 2.5 s^−1^), and **3** (0.6 s^−1^, 0.4 s^−1^, and 0.3 s^−1^) were significantly lower than with *Ps*VAO and *Dc*VAO.

A similar picture was observed for the VAO-mediated oxidation of 4-hydroxybenzyl alcohol (**4**), 3,4-dihydroxybenzyl alcohol (**5**), vanillyl alcohol (**6**), 4-hydroxy-3-bromobenzyl alcohol (**7**), 4-(1-ethanol)phenol (**8**) and 4-hydroxy-3,5-dimethoxybenzyl alcohol (**9**) (see Figure 2B). *Ps*VAO oxidized **5** (2.0 s^−1^), **6** (3.1 s^−1^), and **8** (0.2 s^−1^), *Dc*VAO oxidized **4** (1.2 s^−1^), **5** (0.5 s^−1^), **6** (3.4 s^−1^), **8** (3.5 s^−1^), and **9** (6.3 s^−1^), while the new VAOs were in general less active with these compounds. *Ai*VAO was active with **5** (1.7 s^−1^), **6** (1.0 s^−1^), and **8** (2.5 s^−1^), *Ex*VAO with **5** (0.9 s^−1^), **6** (0.3 s^−1^), and **9** (0.4 s^−1^), *Hi*VAO with **4** (0.2 s^−1^), **5** (0.2 s^−1^), **6** (0.5 s^−1^), **8** (0.3 s^−1^), and **9** (1.2 s^−1^), and *Pv*VAO was only active with **6** (0.3 s^−1^) and **9** (0.1 s^−1^).

For α-hydroxylation (or dehydrogenation) reactions with 4-alkylphenols, a different picture emerged for the reactions with 4-ethylphenol (**10**), 4-ethylcatechol (**11**), 4-ethylguaiacol (**12**), 4-n-propylphenol (**13**), 4-n-butylphenol (**14**), 4-isopropylphenol (**15**) and 4-(1-methylpropyl)phenol (**16**) (see Figure 2C). *Ps*VAO was active with **10** (3.8 s^−1^), **11** (1.2 s^−1^), **12** (7.4 s^−1^), **13** (4.9 s^−1^), **14** (1.5 s^−1^), **15** (1.5 s^−1^), and **16** (0.4 s^−1^). For *Dc*VAO, similar results as for *Ps*VAO were obtained with **10** (2.0 s^−1^), **11** (0.8 s^−1^), **12** (7.6 s^−1^), **13** (2.0 s^−1^), **14** (0.7 s^−1^), **15** (0.4 s^−1^), and **16** (0.2 s^−1^). *Ex*VAO was not able to perform any reaction with 4-alkylphenols, while *Ai*VAO was only active with **15** (0.4 s^−1^). *Hi*VAO was active with **10** (0.2 s^−1^), **11** (0.3 s^−1^), **12** (1.5 s^−1^), **13** (0.5 s^−1^), **14** (0.5 s^−1^), **15** (0.4 s^−1^), and **16** (1.2 s^−1^), and *Pv*VAO was active with **11** (0.5 s^−1^), **12** (0.5 s^−1^), **13** (1.3 s^−1^), **14** (4.9 s^−1^), **15** (2.0 s^−1^), and **16** (0.4 s^−1^). Overall, three out of four new VAOs were active with 4-alkylphenols but they generally did not perform as good as *Ps*VAO and *Dc*VAO. *Pv*VAO was not active with 4-ethylphenol but it is worth noting that with sterically demanding *p*-alkylphenols like **14**, **15**, and **16**, the enzyme performed better than *Ps*VAO and *Dc*VAO, thereby differing from the overall observed substrate scopes.

For the conversion of other substrates of VAOs, phenolic ethers like 4-(methoxymethyl)phenol (**17**), vanillyl ethyl ether (**18**), vanillyl butyl ether (**19**), and phenolic amines like vanillyl amine (**20**) and 4-(1-aminoethyl)phenol (**21**) were tested (see Figure 2D). For the phenolic ethers, *Ps*VAO showed activity with **17** (3.0 s^−1^), **18** (1.8 s^−1^) and to a lesser extent with **19** (0.1 s^−1^), while *Dc*VAO performed good on **17** (0.6 s^−1^), **18** (4.1 s^−1^) as well as **19** (1.5 s^−1^). In comparison, none of the new VAOs showed any activity toward any of these ethers, highlighting again a difference between the first and the second subfamily, as this standard reaction of VAOs is not performed by members of the second subfamily. For the phenolic amines, *Ps*VAO and *Dc*VAO did not show activity with **20** under the tested reaction conditions at pH 7.5, while *Ps*VAO showed some activity with **21** (0.2 s^−1^). Of the new VAOs, only *Pv*VAO showed activity with phenolic amines however, decent for **20** (1.2 s^−1^) and **21** (1.3 s^−1^), and presenting itself as a good candidate for this type of reaction.

Identification of the respective products of the new VAOs was done *via* GC–MS (see Table 1). The oxidation of 4-hydroxybenzyl alcohols and γ-hydroxylation of 4-allylphenols yielded the expected products that have been described for *Ps*VAO (Fraaije et al., 1995) and *Dc*VAO (Eggerichs et al., 2023a). Notable differences were observed in the reactions with 4-alkylphenols. As previously reported, *Ps*VAO mainly produced 1-(4′*-*hydroxyphenyl)alcohols from short-chain 4-alkylphenols and 1-(4′-hydoxyphenyl)alkenes from more bulky 4-alkylphenols (van den Heuvel et al., 1998, 2001; van den Heuvel et al., 2000b). It was also reported for *Ps*VAO that the low yield of (4′-hydoxyphenyl)alkanones was due to the poor further oxidation of the initially formed (*S*)-1-(4′*-*hydroxyphenyl)alcohols, the preferred enantiomeric products of *Ps*VAO (Drijfhout et al., 1998; van den Heuvel et al., 2000a). Here we found that *Pv*VAO successfully catalyzed the oxidation of the initial 1-(4′*-*hydroxyphenyl)alcohol products to the corresponding ketones, suggesting a different stereospecificity. Interestingly, α-hydroxylation did occur in the reactions of *Pv*VAO with the 4-*sec*-alkylphenols **15** and **16**, resulting in the corresponding tertiary alcohols, suggesting a better access of water to the active site than with *Ps*VAO and also *Dc*VAO (van den Heuvel et al., 1998; van den Heuvel et al., 2000b).

**Table 1:** Structures of substrates and the respective products identified *via* GC–MS.

Substrate		Products of the respective VAOs					
No	Structure	*Ps*VAO	*Dc*VAO	*Ai*VAO	*Ex*VAO	*Hi*VAO	*Pv*VAO
1	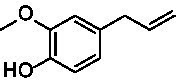	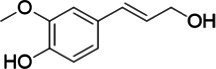	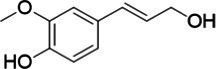	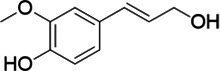	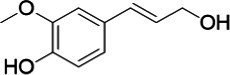	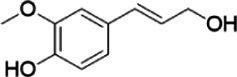	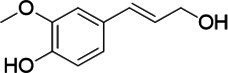
2	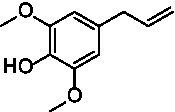	–	–	–	–	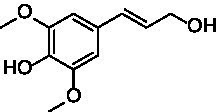	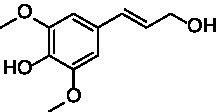
3	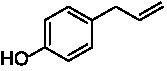	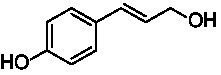	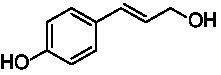	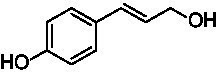	–	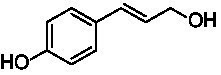	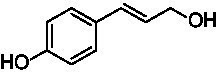
4	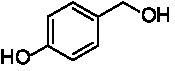	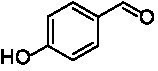	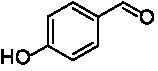	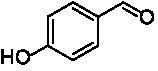	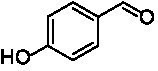	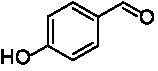	–
5	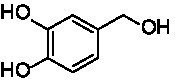	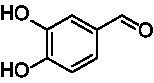	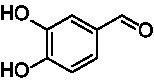	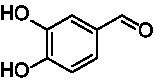	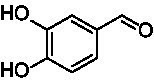	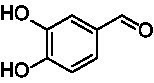	–
6	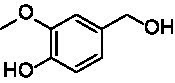	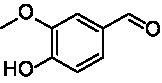	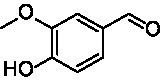	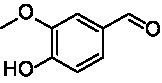	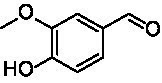	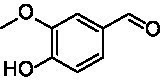	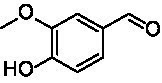
7	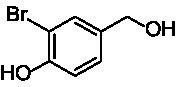	–	–	–	–	–	–
8	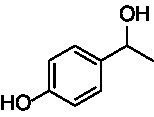	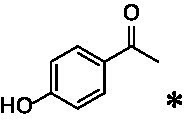	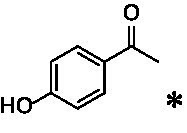	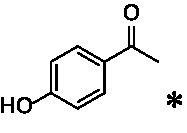	–	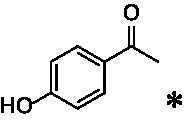	–
9	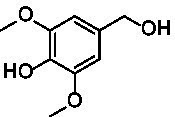	–	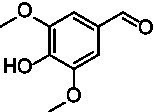	–	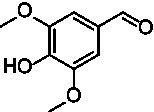	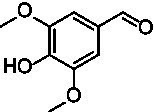	–
10	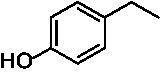	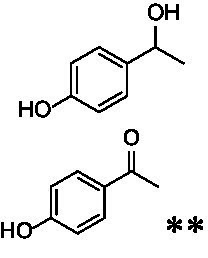	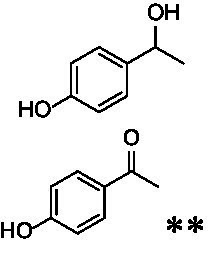	–	–	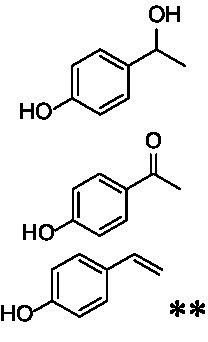	–
11	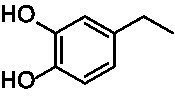	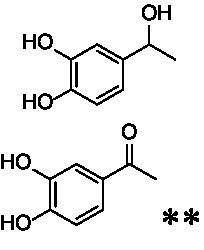		–	–	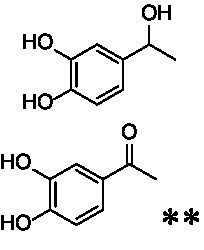	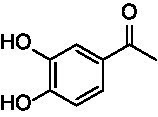
12	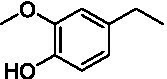	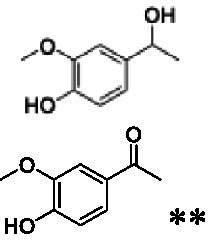	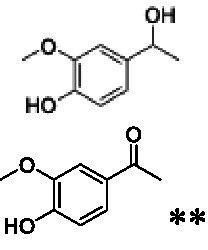	–	–	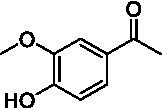	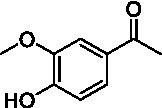
13	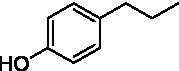	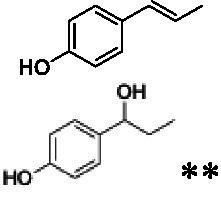	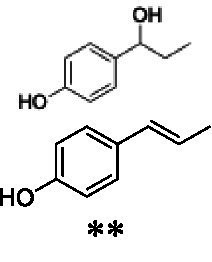	–	–	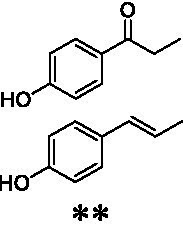	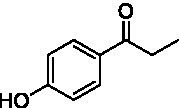
14	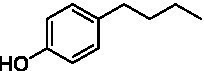	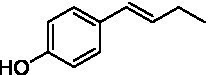	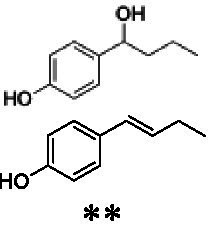	–	–	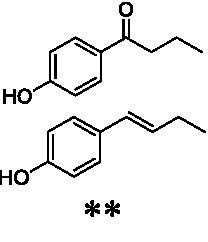	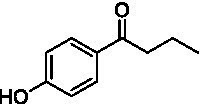
15	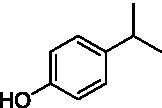	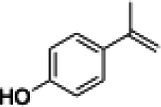	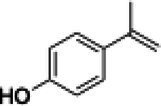	–	–	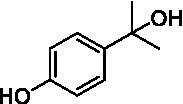	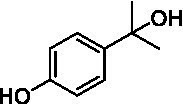
16	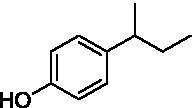	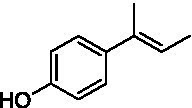	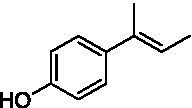	–	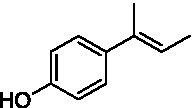	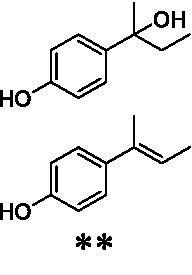	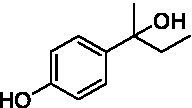
17	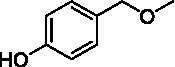	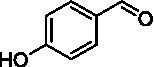	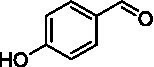	–	–	–	–
18	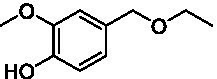	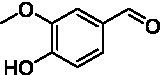	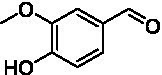	–	–	–	–
19	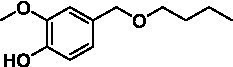	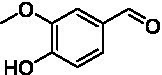	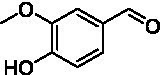	–	–	–	–
20	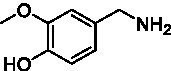	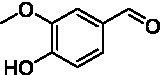	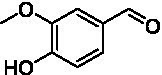	–	–	–	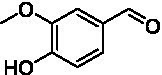
21	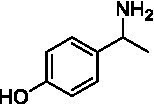	–	–	–	–	–	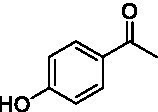

Reactions were set up according to 2.6. The products indicated with a “*” were identified alongside the substrate, which was used in a racemic mixture. Products marked with “**” were identified as a side product. Reactions of *Ps*VAO and *Dc*VAO were run as a comparison for the four new VAOs. Products market with stars are observed only in trace amounts compared to the substrate provided or main product observed.

### 3.3 pH-dependence of oxidative deamination reactions of *Pv*VAO

**Figure 3:**
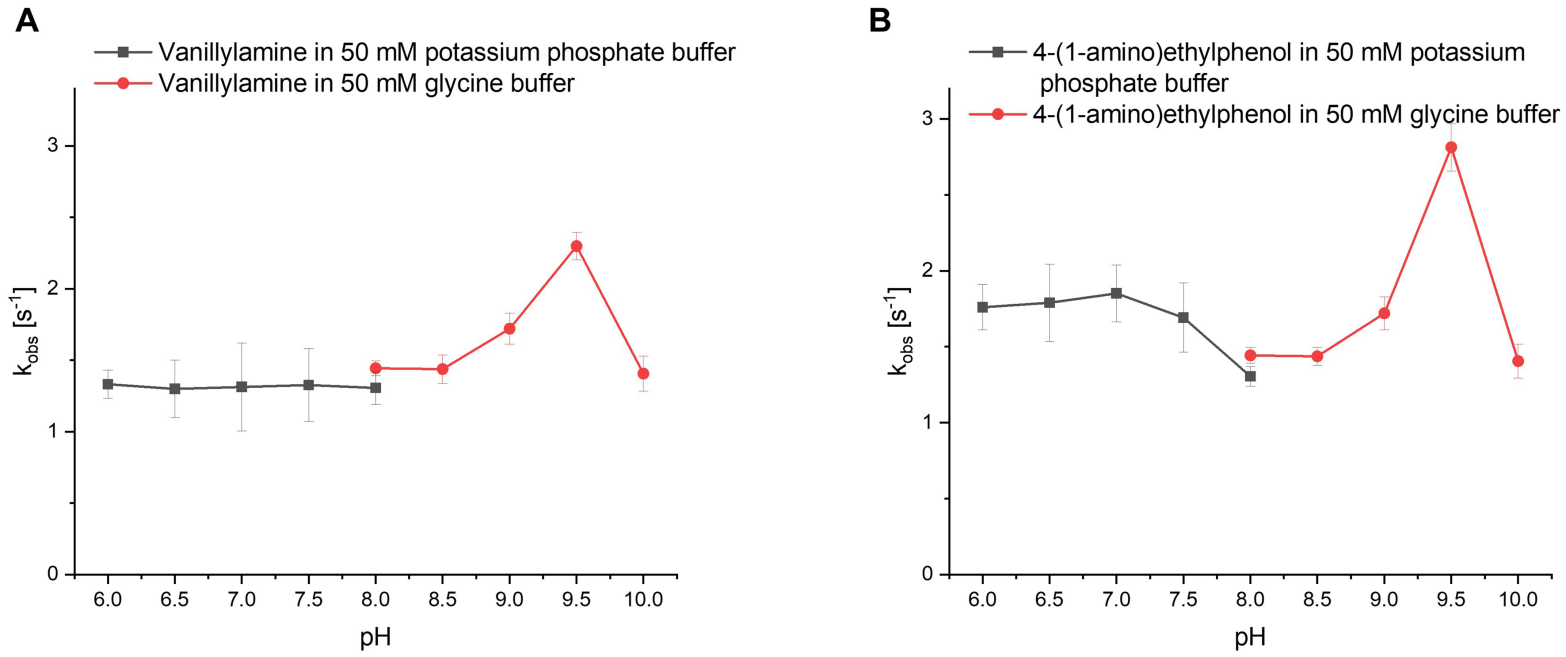
Relative activities for substrates **20 (A)** and **21 (B)** in dependence of pH. Results with 50 mM potassium phosphate buffer and 50 mM glycine buffer are shown in separate lines with pH 8.0 selected as the overlapping pH.

Because *Pv*VAO was found in the initial screening to be rather active with phenolic amines at pH 7.5, the pH-dependency of *Pv*VAO toward **20** and **21** was tested in a pH range from 6.0 to 10.0 using 50 mM potassium phosphate and 50 mM glycine buffer. Britton-Robinson was omitted, as *Pv*VAO did not show any activity using this buffer system. This might be due to an interference of buffer components such as barbital with the applied xylenol orange assay. However, with the glycine buffer system, both substrates **20** and **21** were converted by *Pv*VAO in a pH-dependent manner as described for *Ps*VAO. As seen in Figure 3, both substrates showed an increase in relative activity above pH 8.5, with maximal activity at pH 9.5, which is followed by a steep decline in activity at pH 10.0.

## 4 Discussion

The phylogenetic comparison of the VAOs presented here suggests that VAOs may be divided into two bigger subfamilies. The main distinguishing factor between these two subfamilies is the presence of a loop in the first subfamily, which was shown to be essential for tetramerization of VAO dimers, i.e., octamerization (Ewing et al., 2016), while this loop is absent in the second subfamily. This absence is also seen for the closely related bacterial eugenol oxidases. The first subfamily contains already described VAOs and represents a relatively conserved active site. Clade 6 from the first subfamily is the only clade of this subfamily that shows significant changes in the active site, which may be attributed to the lifestyle of the respective organisms and thereby the changes substrate scope of these VAOs, as proposed earlier (Eggerichs et al., 2023a). It was suggested that the found changes in the A- and P-cluster in *Dc*VAO facilitate the conversion of di*-o-*substituted phenolic substrates, which cannot be converted by other VAOs, like *Ps*VAO. The second subfamily of the VAOs differs significantly in the constitution of the active site, compared to the first subfamily however, distinctive features of clade 6 of the first subfamily are maintained, which would hint at similarities in their substrate scopes, precisely the ability to also convert di-*o*-substituted phenolic substrates, as also observed for *Hi*VAO and *Pv*VAO (Figure 2).

Contrary to our experience with *Dc*VAO (Eggerichs et al., 2023a), the production of four out of the five new VAOs in *E. coli* was achievable in sufficient yields of FAD-loaded proteins for initial characterization, omitting the need for additional help in protein (re)folding. For *Tp*VAO, protein production was not feasible, neither using the conditions used for the other new VAOs, nor the conditions used for *Dc*VAO resulted in the production of a protein that was neither loaded with FAD or could be reconstituted with FAD after purification as it has been described for flavoproteins before (Hefti et al., 2003). For *Pv*VAO, production of an orange protein could be observed, which has been seen with other VAOs before, when produced in *K. phaffii* (Gygli and van Berkel, 2017). The orange color could correspond to the FAD being present in the anionic semiquinone form, which would also be in alignment with previously reported results, where re-oxidation of the FAD was not possible (Gygli and van Berkel, 2017). However, for *Pv*VAO re-oxidation was observed over the course of a few minutes after elution from the His-Trap column, but regardless of that, the protein was inactive. Remarkably, repeating the production and purification of *Pv*VAO under identical conditions, yielded a yellow protein that was active. Precipitation of these four VAOs indicated that these are also covalently bound to their FAD cofactors, as no FAD could be detected in the soluble fraction, while bright yellow sediments could be obtained.

The four VAOs from clade C7 that could be produced also showed activity toward a variety of phenolic substrates. However, it could be observed that within the tested substrates, the scope of performed reactions is more limited compared to already described VAOs and also at lower rates reported for *Ps*VAO and *Dc*VAO. It was also found that except for *Ai*VAO (Figure 2), the second subfamily of VAOs poorly reacts with 4-hydroxybenzylalcohols, which are well established substrates of other VAOs and also the bacterial eugenol oxidases (Alvigini et al., 2021; Eggerichs et al., 2024; Eggerichs et al., 2023b; Ewing et al., 2018; Nguyen et al., 2016). Regardless of the overall lower activities, the new VAOs showed differences in the reactions they can perform, compared to the already described VAOs (Figure 2).

*Pv*VAO presented itself as an exception, being a quite interesting candidate for biocatalytic applications. While it underperformed in standard reactions such as γ-hydroxylation reactions and oxidations of 4-hydroxybenzylalcohols, it performed good in α-hydroxylation reactions of 4-alkylphenols and oxidations of 4-hydroxybenzylamines. For α-hydroxylation reactions, it is worth noting that increasing size and complexity of the *p*-substituent of the 4-alkylphenolic substrates correlated with higher observed activities. As seen in Supplementary Figures S3, S4, this may be related to a slightly bigger active site, based on the modeled 3D-structure of *Pv*VAO. This would then also hint at the possibility that other substrates that have yet not been tested may also be converted by *Pv*VAO. In addition, *Pv*VAO could be used to steer the oxidation of 4-alkylphenols in the direction of the corresponding ketones as these over-oxidation products were only in trace amounts obtained for *Ps*VAO and *Dc*VAO (Eggerichs et al., 2023a; van den Heuvel et al., 2000b). For the latter, as seen in Figure 1 and Supplementary Figure S3, there is only one obvious residue that differentiates the active sites of *Pv*VAO and *Dc*VAO, which is the exchange of L316 (*Ps*VAO numbering) to a glutamate. Thus, it would be of interest to investigate, if this residue in *Pv*VAO is the major contributor to the observed overoxidation of 4-alkylphenols to the corresponding ketones and the improved activity toward 4-hydroxybenzylamines.

As mentioned, the overall low activities for the four new VAOs, with some exceptions for substrates with more complex *p*-substituents may hint at other possible substrates to be naturally converted by these VAOs. This would also be supported by the fact that all of the new VAOs did not perform the cleavage of benzylic ethers, which is performed by *Ps*VAO and *Dc*VAO. For *Ps*VAO, it was even shown that the production of *Ps*VAO in *Penicillium simplicissimum* is induced when grown on 4-(methoxymethyl)phenol, which is also well converted by *Ps*VAO (Fraaije et al., 1997). While the lack of activity toward benzylic ethers is also observed for the bacterial counterparts, the eugenol oxidases, it should also be mentioned here that EUGO from *Rhodococcus jostii* RHA1(*Rj*EUGO) as well as other bacterial homologous, also showed hardly any activity with 4 alkylphenols (Eggerichs et al., 2024; Ewing et al., 2018; Jin et al., 2007), which are still converted by the new VAOs. If this is put in an evolutionary context, the gain of these two functions would place C7 between the EUGOs and C1-C6 of the VAOs, which would also be in alignment with the gain of the octamerization loop of the VAOs for C1–C6 (Supplementary Figures S1, S2).

Like it was shown for *Ps*VAO, *Pv*VAO also displayed a pH-dependent activity profile when performing oxidative deamination reactions. However, the pH range for *Pv*VAO was presented to be narrower than the one of *Ps*VAO, where the maximal activity for *Ps*VAO was reached at pH 10.5, while it was pH 9.5 for *Pv*VAO, which also showed a decline in activity at pH 10.0. While this observation of increasing activity is in alignment with what has been described for *Ps*VAO (van den Heuvel et al., 2001), *Pv*VAO seems to be inactivated at higher pH, which may be related to lower stability at the corresponding pH, compared to *Ps*VAO.

## Data Availability

The original contributions presented in the study are included in the article/Supplementary material, further inquiries can be directed to the corresponding author.

## Footnotes

1
https://blast.ncbi.nlm.nih.gov/Blast.cgi
