# Supplementary material for: Discovery of a new subfamily expands the catalytic versatility of vanillyl alcohol oxidases
Source: Front Microbiol. 2026 Feb 3;17:1769237. doi: 10.3389/fmicb.2026.1769237 (PMC12909556; doi:10.3389/fmicb.2026.1769237)
Supplement: Supplementary file 1 [file Data_Sheet_1.PDF]

Supplementary Material

1 Supplementary Data

1.1 Further phylogenetic comparison of vanillyl alcohol oxidases

A further comparison of the VAOs was performed, in which the loop that was shown to be responsible for the octamerization of VAOs was compared throughout the family. As seen in Figure S1, the residues that constitute the proposed octamerization loop differ between the clades, not only in length but also in sequence, ranging from 14 to17 residues, which are mainly made up of polar residues. For *Ps*VAO, it was shown that the deletion of this loop seems to prevent the octamerization i.e., the formation of tetramers of catalytically active dimers (Ewing et al., 2016). *Dc*VAO was also shown to form a mixture of dimers and tetramers of dimers (Eggerichs et al., 2023). As seen in Figure S1, the loops of *Ps*VAO (C4) and *Dc*VAO (C6) differ in length and sequence. Hence, it may be assumed that this structural element may serve a similar function, whilst differing throughout the individual clades of the VAOs. While the loop can be found for clades C1-C6, it is absent in C7, which makes the latter more similar to the bacterial outgroup (BO). This loop is visualized across clades C1-C6 in Figure S2A, while Figure S2B shows the lack of this loop in C7.

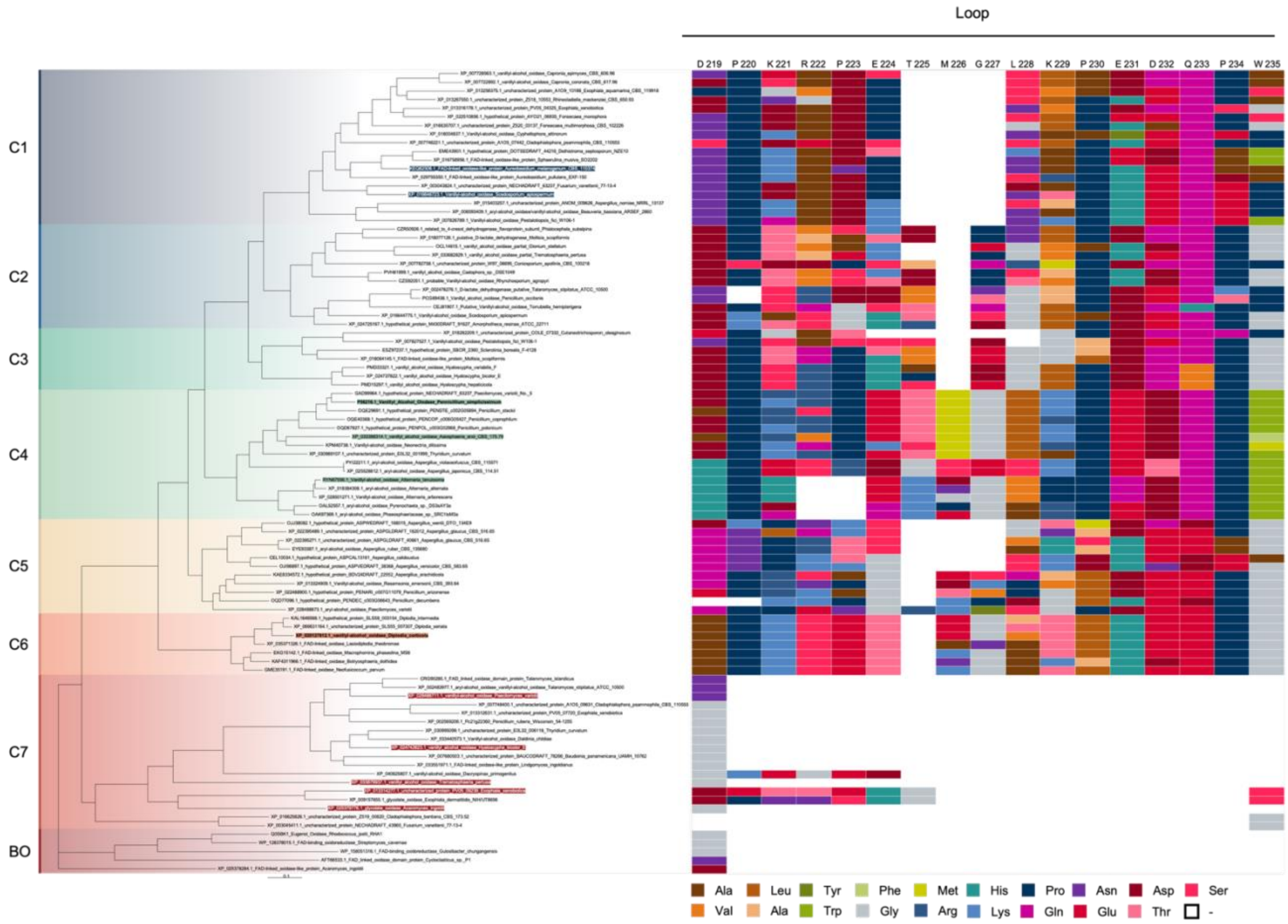

Figure S1: Output of A[2]CA (modified). The generated MSA and phylogenetic tree were used to highlight the diversity of fungal VAOs in their proposed octamerization loops. *Ps*VAO ((NCBI: P56216.1) was used as a reference and the respective positions within the sequence are indicated. The found residues in other VAOs are indicated by color (see legend). Clades C1-C7 are indicated by shaded colors. BO: bacterial outgroup.

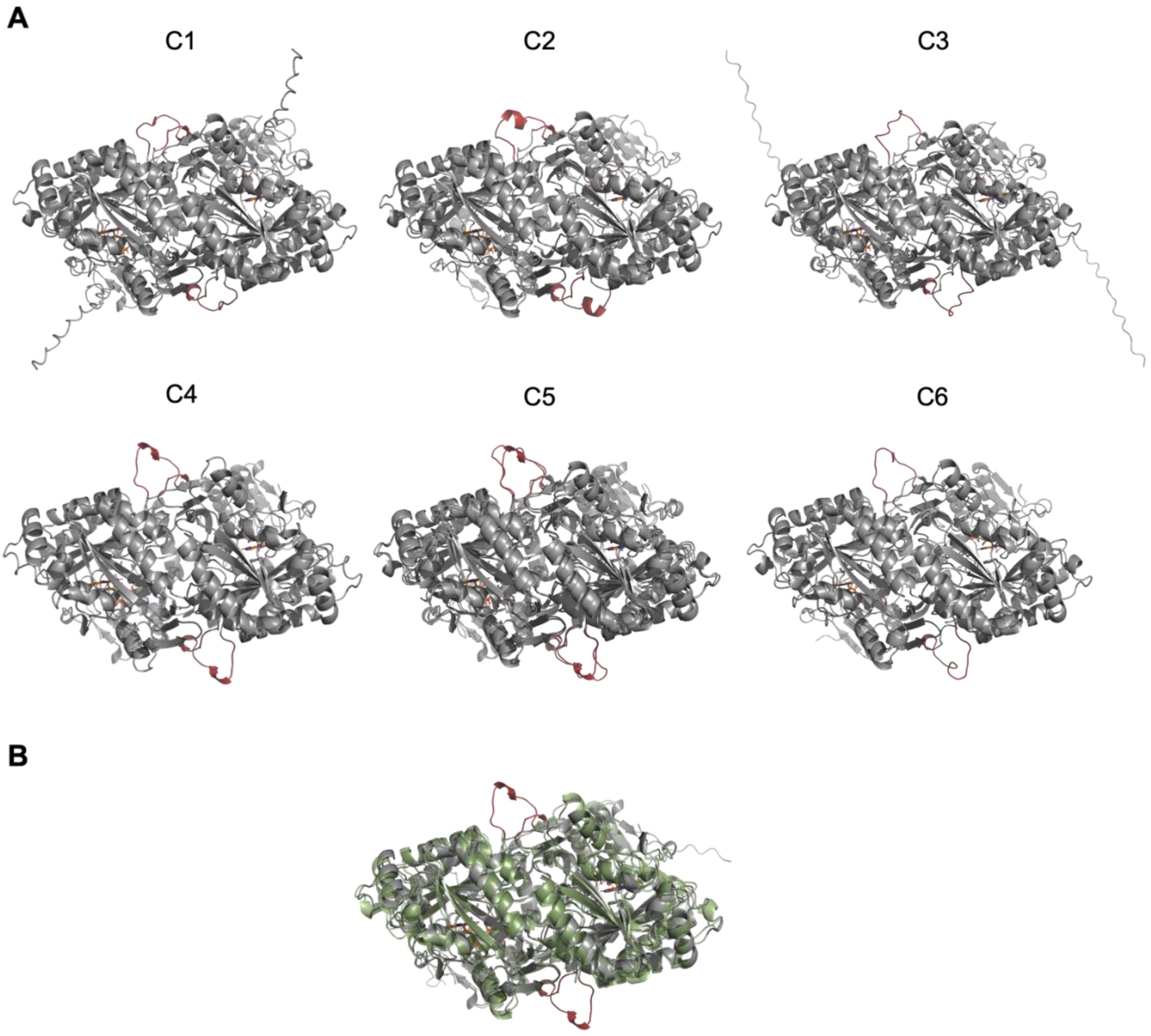

Figure S2: Representation of the loop proposed to be responsible for octamerization, highlighted in dark color. **A:** Representation of the loop in clades C1-C6, based on AlphaFold3 structures (Abramson et al., 2024). For C1, KEQ62306.1 was used, for C2, XP_007782758.1, for C3 PMD33321.1, for C4, *Ps*VAO, using one of the available crystal structures (PDB: 2VAO), for C5, KAE8334572.1, and for C6, XP_020127612.1. **B:** Overlay of *Ps*VAO (grey) and *Pv*VAO (green), showing the lack of this loop in C7.

1.2 Sequences of selected VAOs

Based on the differences that were highlighted by the use of A^{2}CA, five sequences of VAOs from C7 were selected for further investigation. The NCBI accession numbers are given in combination with the designated abbreviations and organisms of origin.

XP_025378284 *Ai*VAO - *Acaromyces ingoldii*

MAPAPTATTLGGEGTTSHRLATTVAGNGAHEGAFKAMVGNKAGVAKNAARYVLAPDVTA
EAFEAFIDEAKQAVGADNVHVNLSASIEDHERGDYLHQPRFHDFFPIDELDKYMASAAIQPA
SVEEVQAIVRAANKHGQPLHTVSIGRNLGYGGSSPRLRGTAILDLKRMNKILEINEKSAYALL
EPGVSYFELYEHLQKTGSNLWVDCPDIGWGSVVGNACDRGAGYTPYGDHWMFRSGLEVV
LPNGELVRTGMGALPDNDTWQCFPYGFGPYHDGIFSQSNLGIVTKMGIWLMPNPGGYEPFLI
TVPRKEDLGPMMDIIGQLRVRMVIQNAPTVRHVLLDAACLKSKAQWTGSDTGAPLGEEEIE
KIARELNLGYWGFYGALYGPEPVRQGIWQAIWGSFQQISGAKSFFEKDVGPESILHSRAKTL
AGIPNLLELDWISWRENGAHNFFSPISAVTGEAAVAQVQLVEEKARKYGFDFMNTLIVGLRE
LHNINCLVYNRESKEERDRMRACIHELIDEAAAKGWGEYRTHNEFMDHVAGTYNFNNGAL
AKLNDTLKDALDPNGILAPGKCGVWPKRYRGRNL

XP_024742623 *Hi*VAO - *Hyaloscypha bicolor* E

MSSKSDIPLLSEKHSGIPLNLLEKAQRAKALIYETRTKETKTRERLPAIPQGIDKSTFIKALDEL
GDQLGTDNVEVNEKPLVDGWYMEHPNTHDMMPILDDEELVASAVVYPSSTEEVQRIVLWA
NKYSIPIFPISMGRNLGYGGAAPRVRGSVVIDLGKRMNKILDINPNDYTCLVEPGVSFYALYE
AIQAKGYDHMWIDTPDLGGGSVIGNTLDRGVGYTPYGDHWACHSGLEVVLPTGEVIRTGM
GALPGNNTWQTFPYGFGPYSDGIFSQSNFGIVTKMGMTLMPNPGGYESFMYAFQKEGDLEA
LIEIIRPLRIGNILENVAQVRHAIQTLAVKGLPRTKYFSGDGPIPEEIVREQLRKMPIGDHTWLY
YGMSYGPPHIRKYKLDIIDQEFKKIPGARKIDPSTLPEDEYFWSRDRIAAGVPDLVELLWVN
WVPNGAHVAFSPVSPIRGADAMKLFNLGKRRHDEFGIDFLPAFCVGLREMHLIVEIVYDKN
DPVKRKAANDCLREMIDDAAKEGYGEYRTHLVLMDQVAGTYSWNDGALMKFNERLKDA
LDPNGILAPGKSGIWPARYRGRGWEMGKSSGGRSEGNGVTPSKNTKL

XP_028486711 *Pv*VAO - *Paecilomyces variotii*

MAKSGITPTPQLGEKHNGIPARLFDKAGHAKSSIWDIATKPEEKKLKKIAIPQGIEETKFFEAL
EDLKNQLGPENVELVEKLVDGWYMENPNTHDGMHILDDEELVASAVVYPGSTADVQKIVL
WANKYRIPIFPISIGRNFGYGGAAPRVRGSVVIDLGRRMNKVLDINPDDCTCLVEPGVTFFAL
YEEIQRRGHKHLWIDVPDLGGGSVMGNTLDRGVGYTPYGDHWTFHSGLEVVLPTGEVIRTG
MGALPNNNSWQIFPYGFGPYSDGIFTQSNFGIVTKLGMALMPDPGGYESYLYTFQKEEDLAP
LVEIIRPLRIANILENVAQLRHVLEQVACSGKPRSTYWKGEGAIPDHVIHEVAKTLSHGDCTW
LYYGMAYGPKEIRQYKLDIIHKEFMKIPGARRIDPTTLPKNDYFWSRDRIAAGIPDLEELAWV
NWHPNGGHIAFSPVSPVRGPDATALWKLAKTRCGQYGLEFFPTYCVGMREMHLIVEIVFDR
NNPTMRQNVDKCIRAMIDDAAKAGYGEYRTHLALMDQIAGTYNWNNNALMRFNEKLKDC
LDPAGILAPGKSGVWPARYRGRGWEIGKEGRQSSEGDGVAPAPGSTRL

XP_013314277_ExVAO - *Exophiala xenobiotica*

MSQEYAPGIPVRVTDYADSVKKRFEENATVPPVKPRKEASEDLPVLPPGVTRQNFNAAIEQL
KSIVDGHVELVDHDLDDGWYLHRPLTHDVFALDEDDYFVNSAICAPGSVEQVQAVVKWAN
QWLIPIYAVSMGRNFGYGGSSARVKGSVVLDMGKRMNRVLELNEKSAFCLLEPGVTYYML
YDEIQKSGKDLWVDVPDLGGGSVMGNSLDRGVGYTPYGDHFGMHCGMEIVLPDGEVIRTG
MGALPDETTEHGSSNTWQLFPYGFGPYSDGIFTQSNYGIVTKMGFWLMPNPGGHQTFQITFP
REDDLHDIIEIMRPLRIKNIIQNTPHLRHIMQEASVYGNKKSYWPHDGPIPHDKIDEIIVPKFEW
IGNFRWILYLCVYGPDVVRKANIQVITEEFSKIPGAKVMFPEETPDYSYLRSRVNIYAGTPDL
RELDWVMWLDNGSHTAFSPISPLTGKDAEKQYQLTKRLHQKWGFDYFPTFCPGWREMHHI
VMIIYDRGDPDSKHRARCLMEELVAEGAKVGMGEYRTHLALQDQVMGTYSWNNHALLRM
NNKIKDTLDPNGIYAPGKSGIWGRCWDNRDSAHPSRVSASSQEPQGRSTLGRLSSQVSDQLS
KSRL

XP_033679937 TpVAO - *Trematosphaeria pertusa*

MPASIPSTASPAGRPKKIERPIWHGTVLSQHPSSPYISKTRDERPIVLPPHTSESAFETALRELR
ELLGEEWVKCNDVALVDGDYHAVPLSHDAYHILEQDDLVPSAVCWPKDTEQVATVVKWA
NRHKIPIWPISIGRNLGYGGSAPRVRGSVVVDLGRRMNRVLDVSEQNAACLVEPGVQYITLY
EHLQEIGLGNKLWIDVPDLPGGSVMGNALDRGVGYTPHGDHWSQHCGLEVVLPDGDIVRT
GMGALPGTDCWQLFPYGFGPYHDGIFSQSNYGIVTKMGVWLMPNPGGIRPFMATFPREEDL
AAAVDALRPLMVQKIVGNVPCLRLGVQDATMYHKKTEFPLRENGLIDRKKAQTMIDKLGT
GAWVFYGALYGPDEITAPKFDYIKKALSVVSGAKFYERKDVPSNHYLNDYAKFTAGIPTWR
ELDRMQFIPNASHLFFAPISAIDGKDALRQSHMCRNRMEEYGFDYLGTFFIGHRAMHHVISF
MYDKTDPEMKKRALSCIRALIEDAAKIGVGEYRTHLALQDQVAGTYNWGDGATMRLNEKL
KDALDPNGILQPGRSGIWPKRYRGQGWELKGGEERSDILFKRSAGRL

1.3 Comparison of active sites based on models of the selected VAOs

Using AlphaFold3, models of the five selected VAOs were generated as dimers with one FAD per protomer. Using YASARA, the covalent bond between the corresponding histidine from the H-cluster and the FAD was introduced into the respective models as well as docking in 4-ethylguaiacol as a putative substrate, followed by a MD simulation for 10 ns using the Amber13 force field. The active sites of the VAOs were then visualized and investigated in PyMOL and CavitOmiX (Innophore, Graz, Austria) was used to map the cavities that form the active sites and to also compare their respective hydrophobicities. As mainly the W- and A-clusters were considered for the comparison of the new VAOs to *Dc*VAO, these two clusters are compared in Figure S3. Figure S4 shows a comparison of the active sites of *Pv*VAO, *Ps*VAO and *Dc*VAO to highlight possible differences with regard to size and hydrophobicity, using CavitOmiX. For the cavities, it can be seen that the sizes of the three VAOs differ, with *Dc*VAO having the smallest and also most hydrophilic active site in the region of the A-cluster (E466), followed by *Ps*VAO where the cavity protrudes into the substrate tunnel, however still limited in space in the regions of the A- and W-clusters. *Pv*VAO displays the biggest active site, with its cavity extending between the A- and W-cluster where it ends in a hydrophobic stretch, while *Pv*VAO remains as hydrophilic in the part of the A-cluster that is shared with *Dc*VAO (E492).

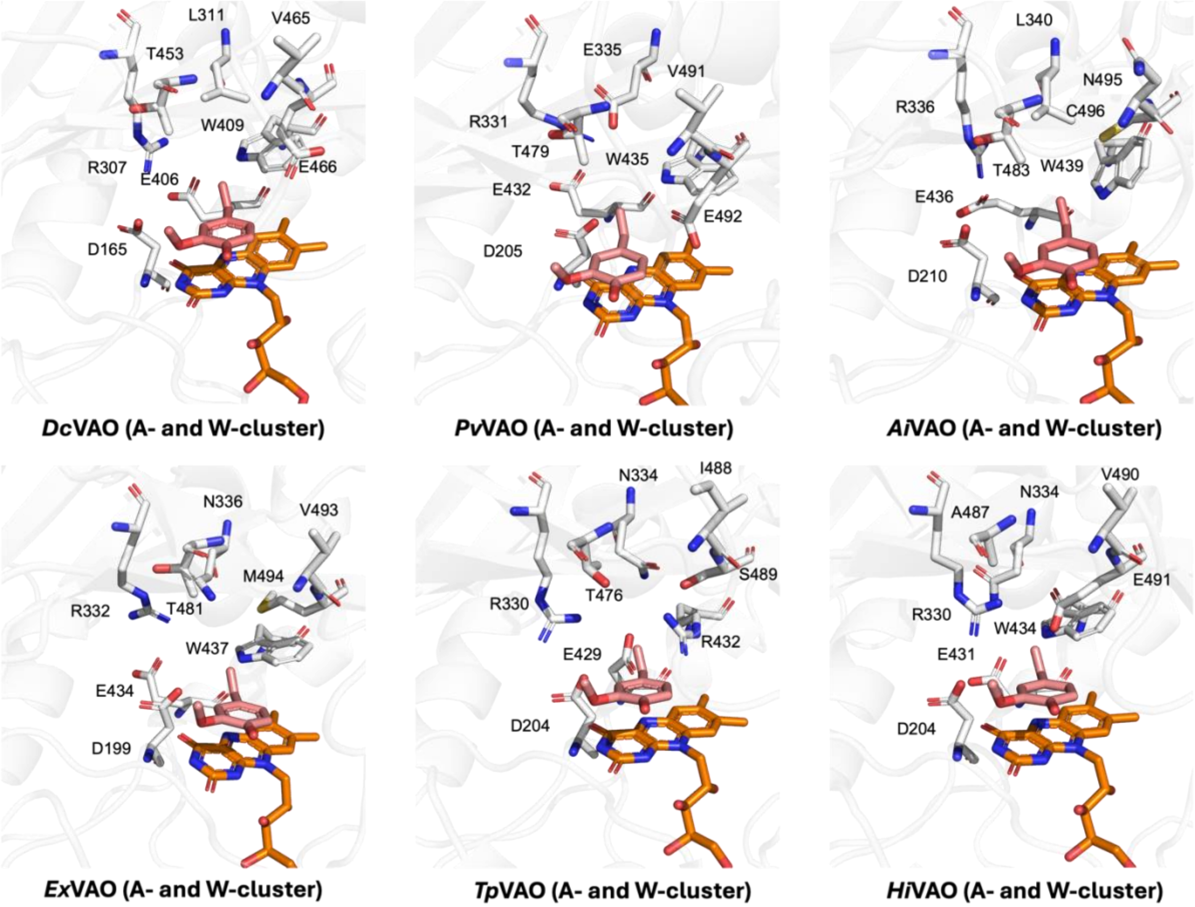

Figure S3: Comparison of the active sites of the five selected VAOs to *Dc*VAO. Shown in stick representation are the residues that constitute the W- and A-clusters.

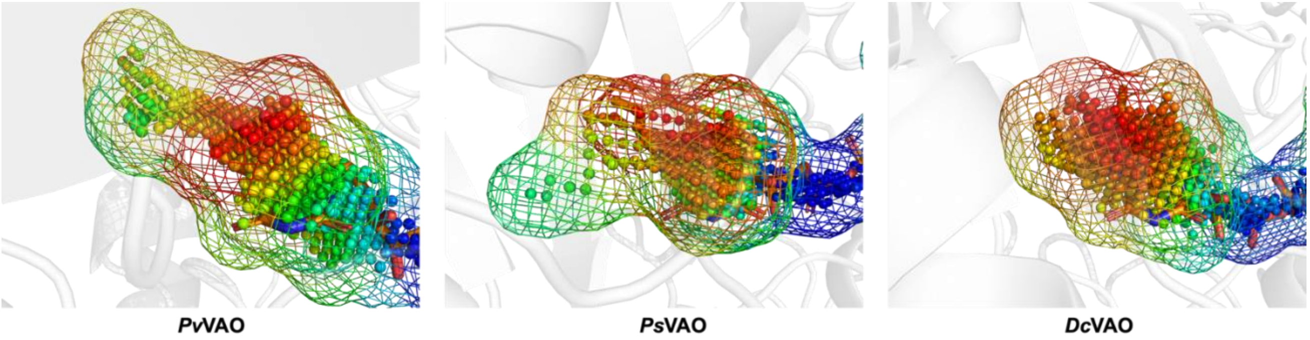

Figure S4: Comparison of the active sites of *Pv*VAO,*Ps*VAO and DcVAO. Highlighted are the cavities that were identified using CavitOmiX, based on hydrophobicity.

1.4 Primers

Table S1: List of primers used for cloning of *vao* genes into pET28a for heterologous protein production in *E. coli* NiCo21 (DE3).

Fragment:	Primer name:	Sequence (5'-> 3'):
pET28a Backbone	pET28a BB for	gcggccgcactcgagc
	pET28a BB rev	Atggctgccgcgcg
*Ai*VAO	pET28a AIVAO for	cagcagcggcctggtgccgcgcggcagccatATGGCCCCGGCACCTACAG
	pET28a AIVAO rev	tggtggtggtgctcgagtgcggccgcTTAAAGGTTACGCCCCCGATAACG
*Pv*VAO	pET28a PvVAO for	ggcctggtgccgcgcggcagccatATGGCTAAGTCGGGTATCACACCAAC
	pET28a PvVAO rev	tggtggtggtgctcgagtgcggccgcTTATAACCGTGTTGACCCTGGGGC
*Tp*VAO	pET28a TpVAO for	acagcagcggcctggtgccgcgcggcagccatATGCCGGCATCCATCCCG
	pET28a TpVAO rev	gtggtggtggtggtggtgctcgagtgcggccgcTTACAGCCGGCCTGCGC
*Hi*VAO	pET28a HiVAO for	tggtgccgcgcggcagccatATGTCTTCGAAATCTGATATCCCGTTATTG
	pET28a HiVAO rev	tggtgctcgagtgcggccgcTTACAATTTTGTGTTTTTGGATGGGGTCAC
*Ex*VAO	pET28a ExVAO for	agcggcctggtgccgcgcggcagccatATGAGTCAAGAATACGCCCCCGG
	pET28a ExVAO rev	ggtggtgctcgagtgcggccgcTCACAAGCGAGATTTGCTTAACTGATCG

1.5 Protein yields and UV/Vis spectra

Table S2: Yields of the respective VAOs per liter of culture after purification via Ni-NTA affinity chromatography. The amount of protein was determined by means of determining the absorption at 441 nm, specific for the covalently bound FAD-cofactor, as described earlier (Jin et al., 2007). The yield of *Tp*VAO was determined by measuring the absorbance at 280 nm using an Implen NanoDrop NP80 (Munich, Germany). As *Tp*VAO could not be purified as a FAD-loaded protein and could also not be reconstituted with FAD as described earlier (Hefti et al., 2003), it was not considered for further investigation.

Protein	Yield [mg/l culture]
*Ai*VAO	15.1
*Ex*VAO	22.1
*Hi*VAO	9.3
*Pv*VAO	20.5
*Tp*VAO	3.9

It was also tested, if the four VAOs are also covalently bound to their FAD cofactor. For this, each of the VAOs was precipitated with trifluoroacetic acid (TFA) as published earlier (De Jong et al., 1992). Changes to the published method were the exchange of trichloroacetic acid (TCA) to TFA (500 mM) and that all four proteins were adjusted to a concentration of 12.5 µM. After sedimentation of the denatured proteins, no FAD could be detected photometrically, but all four sediments were colored bright yellow, indicating that all four VAOs are covalently attached to their FAD cofactor.

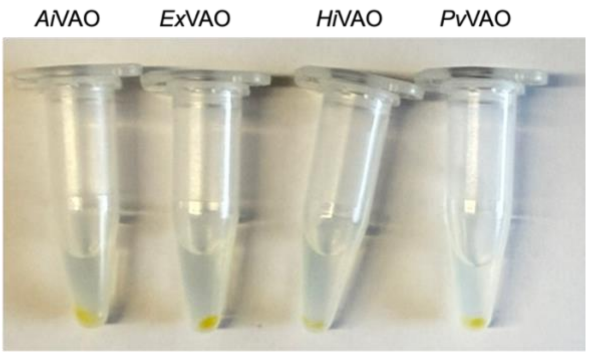

Figure S5: Picture of the sediment and supernatant of the four VAOs after denaturation as described earlier (De Jong et al., 1992).

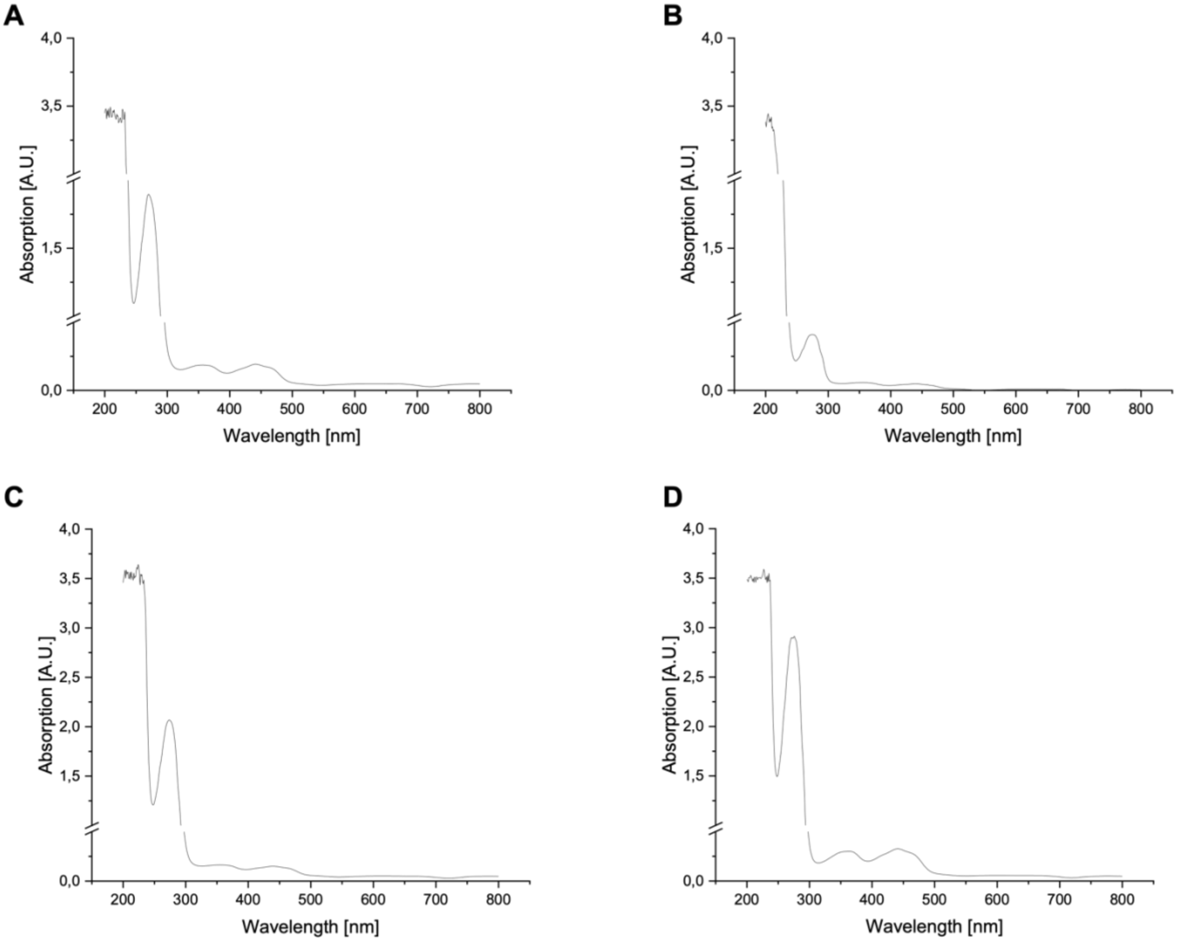

Figure S6: UV/Vis spectra of the four new VAOs that could be purified as FAD-loaded proteins. Spectra were recorded immediately after protein purification without further concentration and buffer exchange. Blanks were subtracted from the spectra. **A:** *Ai*VAO. **B:** *Ex*VAO. **C:** *Hi*VAO. **D:** *Pv*VAO.

2 References

Abramson, J., Adler, J., Dunger, J., Evans, R., Green, T., Pritzel, A., Ronneberger, O., Willmore, L., Ballard, A. J., Bambrick, J., Bodenstein, S. W., Evans, D. A., Hung, C.-C., O'Neill, M., Reiman, D., Tunyasuvunakool, K., Wu, Z., Žemgulytė, A., Arvaniti, E.,…Jumper, J. M. (2024). Accurate structure prediction of biomolecular interactions with AlphaFold 3. *Nature*. https://doi.org/10.1038/s41586-024-07487-w

De Jong, E., van Berkel, W. J. H., Zwan, R. P., & Bont, J. A. M. (1992). Purification and characterization of vanillyl-alcohol oxidase from *Penicillium simplicissimum*. A novel aromatic alcohol oxidase containing covalently bound FAD. *European Journal of Biochemistry*, *208*(3), 651-657. https://doi.org/10.1111/j.1432-1033.1992.tb17231.x

Eggerichs, D., Weindorf, N., Mascotti, M. L., Welzel, N., Fraajie, M. W., & Tischler, D. (2023). Vanillyl alcohol oxidase from *Diplodia corticola*: Residues Ala420 and Glu466 allow for efficient catalysis of syringyl derivatives. *Journal of Biological Chemistry*, *299*(7), 104898. https://doi.org/10.1016/j.jbc.2023.104898

Ewing, T. A., Gygli, G., & van Berkel, W. J. H. (2016). A single loop is essential for the octamerization of vanillyl alcohol oxidase. *The FEBS Journal*, *283*(13), 2546-2559. https://doi.org/10.1111/febs.13762

Hefti, M. H., Milder, F. J., Boeren, S., Vervoort, J., & van Berkel, W. J. H. (2003). A His-tag based immobilization method for the preparation and reconstitution of apoflavoproteins. *Biochimica et Biophysica Acta (BBA) - General Subjects*, *1619*(2), 139-143. https://doi.org/https://doi.org/10.1016/S0304-4165(02)00474-9

Jin, J., Mazon, H., van den Heuvel, R. H. H., Janssen, D. B., & Fraaije, M. W. (2007). Discovery of a eugenol oxidase from *Rhodococcus* sp. strain RHA1. *FEBS Journal*, *274*(9), 2311-2321. https://doi.org/10.1111/j.1742-4658.2007.05767.x
